# Unsupervised Person Re-Identification with Attention-Guided Fine-Grained Features and Symmetric Contrast Learning

**DOI:** 10.3390/s22186978

**Published:** 2022-09-15

**Authors:** Yongzhi Wu, Wenzhong Yang, Mengting Wang

**Affiliations:** 1 School of Information Science and Engineering, Xinjiang University, Urumqi 830046, China; 2 Key Laboratory of Multilingual Information Technology in Xinjiang Uygur Autonomous Region, School of Information Science and Engineering, Xinjiang University, Urumqi 830046, China

**Keywords:** person re-identification, attention, fine-grained feature, contrast learning, unsupervised learning

## Abstract

Unsupervised person re-identification has attracted a lot of attention due to its strong potential to adapt to new environments without manual annotation, but learning to recognise features in disjoint camera views without annotation is still challenging. Existing studies tend to ignore the optimisation of feature extractors in the feature-extraction stage of this task, while the use of traditional losses in the unsupervised learning stage severely affects the performance of the model. Additionally the use of a contrast learning framework in the latest methods uses only a single cluster centre or all instance features, without considering the correctness and diversity of the samples in the class, which affects the training of the model. Therefore, in this paper, we design an unsupervised person-re-identification framework called attention-guided fine-grained feature network and symmetric contrast learning (AFF_SCL) to improve the two stages in the unsupervised person-re-identification task. AFF_SCL focuses on learning recognition features through two key modules, namely the Attention-guided Fine-grained Feature network (AFF) and the Symmetric Contrast Learning module (SCL). Specifically, the attention-guided fine-grained feature network enhances the network’s ability to discriminate pedestrians by performing further attention operations on fine-grained features to obtain detailed features of pedestrians. The symmetric contrast learning module replaces the traditional loss function to exploit the information potential given by the multiple samples and maintains the stability and generalisation capability of the model. The performance of the USL and UDA methods is tested on the Market-1501 and DukeMTMC-reID datasets by means of the results, which demonstrate that the method outperforms some existing methods, indicating the superiority of the framework.

## 1. Introduction

Person re-identification (Re-ID), which refers to the concatenated matching of images of the same identity across different cameras, is a basic image-retrieval task [1]. The advantage of this technique is that it can be used to identify specific people without acquiring clear facial images and allows the use of crossed cameras for people-trajectory reconstruction and target-detection identification. The features it acquires can be used as complementary features for people detection and face recognition, helping to improve and enhance the performance of target-detection and face-recognition systems. It is also important to use person re-identification to monitor case movement routes, especially during global epidemic crises, in order to provide reliable data for epidemic prevention services and to achieve precise prevention and control.

The field of person re-identification is currently dominated by supervised methods, which rely on labelled pedestrian data for model training. The core algorithms commonly used are divided into feature-based learning methods, which focus on learning the invariant features of pedestrians and represent a kind of strongly supervised information, and metric learning methods, which focus on learning similarity measures of features and represent a kind of weakly supervised information, whose algorithm performance still dominates in mainstream datasets [2]. However, in real life, pedestrians are in complex environments and pedestrian data collected under intelligent surveillance are highly susceptible to lighting, foreign-object occlusion or target-detection algorithms, so models trained on specific publicly available datasets are not representative of the actual situation. Moreover, labelling the pedestrian data in each training set under different cameras often requires significant human and financial resources, making supervised approaches difficult to apply to real-life situations and having significant limitations. Therefore, how to use large-scale unlabelled image data to train a model with a stronger generalisation capability to cope with various complex surveillance scenarios is the main research direction in the field of person re-identification in the future.

The general unsupervised person-re-identification task is studied in two main stages: the first stage is the feature-extraction stage, where the extracted pedestrian features directly determine the performance of the network model; the second stage is the unsupervised learning stage, which relies heavily on the features obtained in the feature-extraction stage, and some invalid features will directly affect the subsequent clustering effect. However, some current algorithms, in their first stages, rarely improve on the feature extractor [3,4]. The most intuitive method for selecting pedestrian representation features is to directly extract a global feature map of the pedestrian. Relying on global features alone often fails to accurately identify pedestrians in the presence of occlusion, misalignment and background interference. In addition, some key local features (such as carry-ons and body parts such as the pedestrian’s face or limbs) are not clearly observed due to interference from factors such as low camera resolution and illumination.

Meanwhile, in the second stage, the traditional classification loss and ternary loss have significant drawbacks. The parametric classification loss has to be implemented with the help of a fully connected classification layer; however, the fully connected layer often has a relatively large number of parameters and is not used at all in the inference stage of the model. Moreover this type of loss cannot learn sufficiently for images with fewer categories, and the overall benefit is low. Ternary loss can only handle relationships between three samples at a time, with too many samples required, leading to too slow convergence of the loss function and lengthening the model training time [5,6,7]. So, in order to optimise the metric learning stage and improve the model’s ability to discriminate between features, we use contrast learning. Apart from this, previous methods simply use cluster centres or all instance features for contrast learning, where the cluster centres simply sum all features in each class to take the mean value without considering the correctness of the samples in the class, and there may be sample features that are clustered incorrectly, while direct contrast learning using all instance features will put all features into the storage unit for updating. The former will amplify the clustering noise, and the latter will seriously affect the update speed of the storage unit.

**Figure 1:**
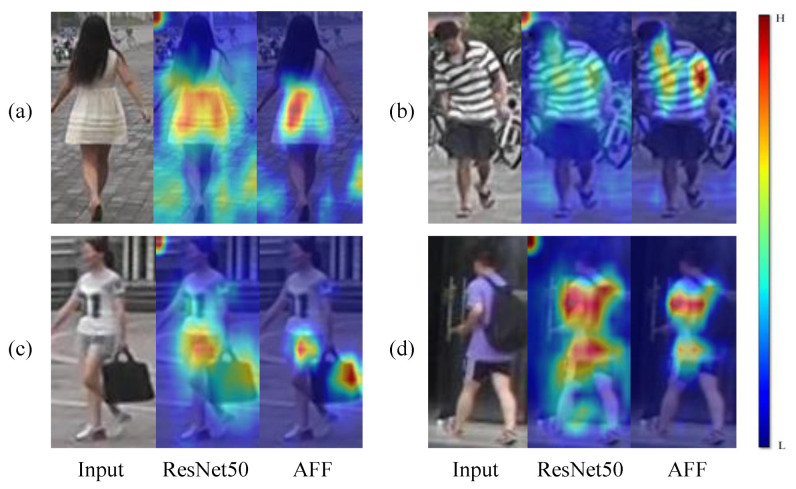
In this paper, the pedestrian image features are visualised in the baseline model (ResNet50) and the attention-guided fine-grained feature network (AFF) in Grad_CAM, and four pedestrian images (**a**–**d**) are selected for visualisation and comparison analysis. It can be seen from this that using the proposed AFF model in this paper can better focus on pedestrian information compared to ResNet50, focusing on pedestrian details such as backpack, clothing colour or accessories, etc. This can better distinguish some similar pedestrians, improve the accuracy of retrieval and verify the effectiveness of the model.

To address the above existing problems, in the first stage, as shown in Figure 1, we propose an attention-guided fine-grained feature network (AFF) by combining fine-grained features and two attentional mechanisms. It will guide the network to discover salient cues at different scales sequentially, from coarse to fine, in order to obtain a comprehensive and complementary perception. Specifically, we adopt hybrid attention to focus on the pedestrian in the picture, reduce the interference brought in by the background and obtain the global features of the pedestrian. The processed features are then segmented on the channel, and the channel attention is employed to mine semantic information. By combining spatial local-to-local and semantic concept-to-concept matching, we are able to establish a fine-grained feature-fusion approach with attention to achieve generalised and distinguishable representations and improve the ability of the neural network to extract more discriminative features. In the second stage, we propose a symmetric contrast learning (SCL) method using mean features combined with hard sample features instead of the traditional loss function. Moreover the hard sample features introduced can be well mined for pedestrian-distinguishing information and a balance loss is introduced to ensure that the network is updated in a more stable manner. Symmetric contrast learning can make full use of information from multiple samples that complement each other to improve the robustness of this stage against noise robustness.

In summary, the main contributions of this paper are as follows:

1. For the feature-extraction stage, i.e., how to obtain “effective” people features from the model and avoid the interference of background and other noise in the people images, so as to prepare for better clustering, this paper introduces an AFF network. By combining the fine-grained features of people images with the attention mechanism, we can improve the distinguishability of people features and thus enhance the discriminative power of the model.
2. For the unsupervised learning stage, i.e., how to reduce the influence of “invalid” data in the clustering process, reduce the clustering error and improve the robustness of the model, this paper introduces a SCL method. Instead of adopting a single feature in the selection of clustering representatives in the storage unit, a combination of mean features and hard sample features is adopted to design a symmetric contrast loss to improve the generalization ability of the model.
3. Combining the methods proposed in the two stages, we construct an unsupervised person-re-identification framework AFF_SCL and conduct performance tests on the Market-1501 and DukeMTMC-reID datasets from both the Unsupervised Learning(USL) and Unsupervised Domain Adaptation(UDA) methods. Additionally, the results show the superiority of the person-re-identification framework designed in this paper.

## 2. Related Work

### 2.1. Supervised Person Re-Identification

When extracting pedestrian features in person-re-identification tasks, using only global features to train the model no longer improves the model’s ability to discriminate between complex samples. As research continues, researchers have started to apply finer features and learn local fine-grained features [3,4,8,9,10,11,12]. From fine-grained features, the global feature information is somehow decomposed into local features, and the decomposed features are taken separately for training to learn finer feature information.

The different ways of dividing feature regions can be divided into three categories; **the first one** is spatial chunking based on information such as body keypoints [3,4,8]. In 2018, Sun et al. [8] proposed a part-based convolutional chunking network that spatially chunks the image uniformly to obtain fine-grained features and then applies different loss functions to different parts. Most of the subsequent spatial chunking has also been analysed based on this method. However, the disadvantage of image spatial binning is that this method requires checking the alignment of key parts of the pedestrian’s image. If the key parts of the body do not correspond to the same location after the pedestrian image is chunked, there is no guarantee that the feature information at the corresponding location will be extracted, so there will be non-convergence in the learning process of the model. **The second one** is the segmentation of channel information based on mapped depth features [9,10]. Chen et al. [9] validated an approach to image channel segmentation that does not extract the local features of specific body parts, but instead typically divides features that have been represented by a deep network into a series of bands or unordered blocks in the channel dimension and forms independent channel groups. The semantic concepts of each channel group can be correlated and local features learned separately. The extracted pedestrian features are divided into chunks according to the channel size and then processed. As the Convolutional Neural Network (CNN) progresses, channel segmentation allows for semantic concept-to-concept matching (e.g., with or without a hat, etc.), with the final layer being more abstract, and the output of the channel being more semantic in character, potentially corresponding to concepts such as hair colour, body type, etc. This semantic information is not only useful for discriminating pedestrians, especially pedestrian features that are extremely similar. Moreover, it avoids the problem of feature misalignment and reduces the omission of detailed information in key areas. Moreover, this semantic information shows strong generality in cross-domain person-re-identification tasks. In contrast, image channel segmentation can also suffer from partial semantic gaps, which we use a channel attention approach to improve. **The third one** uses attention mechanisms to focus on local regions of interest [11,12]. The attention model mitigates misalignment by discovering salient regions in the image, while the learned features are more robust and can effectively help deep neural networks focus on the information of interest. For fine-grained pedestrian analysis tasks, Liu et al. [12] proposed a HydraPlus-Net architecture based on a multi-directional attention module, which was able to capture multiple attention features from the lower to the semantic layer. The final feature representation of pedestrian images is enriched by incorporating a multi-scale selection of attentional features. As the focus varies with the scale of the image, the attention mechanism may eliminate useful additional information. However, through research, this information can be preserved by fine-grained features.

These three methods can make it easier for the network to extract finer and more effective pedestrian features. Channel segmentation allows the neural network to learn more local features, while then combining the attention mechanism to guide the neural network to locate the most important regions in the image and extract secondary semantic information. The complementary nature of attention and fine-grained features can effectively preserve the problem of missing information.

### 2.2. Unsupervised Person Re-Identification

In general, researchers have divided person re-identification into two lines of research: Unsupervised Learning (USL) person re-identification and Unsupervised Domain Adaptive (UDA) person re-identification, which have achieved excellent performance on commonly used publicly available datasets and even outperformed supervised methods on some individual datasets.

#### 2.2.1. Unsupervised Learning Person Re-Identification

The idea of unsupervised-learning person re-identification is usually to use a pseudo-label-generation approach to train the model directly using unlabelled datasets in the target domain, which fully explores the distribution of data in the target domain to learn discriminative human features, and many researchers have used traditional clustering algorithms to generate pseudo labels.

Initial researchers combined K-Means clustering methods and fine-tuned models to achieve this [13,14]. Later Lin et al. [5] proposed a Bottom-Up clustering approach considering the relationship between CNNs and individual samples. Since each pedestrian image is considered to be a different class, the features extracted from the network during training are clustered using bottom-up clustering to ensure the similarity of identity information within the same class and the difference of identity information between different classes. Fu et al. [15] proposed a natural self-similarity grouping method, which independently groups the target domain images based on three cues: full-body, upper-body and lower-body. According to the corresponding groupings, the corresponding pseudo labels are assigned using Density-Based Spatial Clustering of Applications with Noise (DBSCAN) and trained iteratively to continuously explore the potential similarities between global and local.

However, in order to reduce the noise error generated in the pseudo-label-generation process and improve the quality of the pseudo label, some researchers conducted further research. Zeng et al. [6] used Hierarchical Clustering with hard-batch Triplet loss to reduce the effect of difficult samples and mine the similarity of not-easily-distinguishable samples in the target domain with the help of hierarchical clustering to generate high-quality pseudo label. To solve the problem of being affected by noisy labels and feature changes caused by camera shifts, Yang et al. [16] used a dynamic symmetric cross-entropy loss algorithm to deal with noisy samples and proposed a perceptual meta-learning algorithm adapted to cross-camera shifts to cope with camera shifts, which can effectively solve the feature change problem.

In addition to the above-mentioned use of clustering algorithms, some researchers have generated pseudo label with the help of some discriminative information, such as pedestrian-attribute information or identity information to generate pseudo labels to update unlabelled data in the target domain. Yang et al. [17] extracted block samples from the feature map of pedestrian images with the help of a chunked discriminative feature-learning network to generate pseudo labels to learn differentiated block features, demonstrating those local features. Xuan et al. [18] proposed a pseudo-label-generation method based on intra–inter camera similarity and decomposed the sample similarity computation into two stages: intra-camera computation and inter-camera computation.

Clustering-algorithm-based pseudo-label-generation has now become the main method for creating pseudo labels in unsupervised learning person-re-identification scenarios. This approach is simple to implement and can approach the performance of supervised learning methods. Generating pseudo labels based on auxiliary information may consider whether the auxiliary information and the identity information are strongly correlated, and this method is less used.

#### 2.2.2. Unsupervised Domain Adaptive Person Re-Identification

In recent years, unsupervised domain adaptation has become an important research topic in the field of deep learning, and some work has also used unsupervised domain adaptation to solve cross-domain person-re-identification tasks. Traditional domain adaptation methods [19,20] for solving inter-domain differences assume that the labelled source and unlabelled target domains share the same class and use asymmetric multi-task learning methods for dictionary learning to solve the domain-adaptation problem. Some deep learning methods are now well suited to eliminate data discrepancies between source and target domains [7,21,22,23].

**The first approach** is based on image-style migration. Wei et al. [24] and Deng et al. [25] reduced the domain gap between different datasets by designing a pedestrian-migration generative adversarial network to transfer the source domain image style to the target domain image. Chen et al. [26] utilised an instance-based context-mapping approach to input arbitrary source domain data into a GAN network and target domain images, generating images with the same identity as the source domain and the same context as the target domain, never reducing the differences between the inter-domain data due to the image context. The approach based on image-style migration is highly dependent on the quality of the images generated by the generative adversarial networks; so, one of the research challenges is to improve the quality of the generated images.

**The second approach** is a camera-awareness-based approach. To mitigate the appearance of pedestrians under different cameras that may be affected by environmental factors such as viewpoint and illumination, camera-aware approaches are used to reduce the feature-space domain shifts caused by crossed cameras, which can better capture the similarity relationships between and within cameras. At this stage, Qi et al. [27] proposed a camera-aware domain adaptive method based on adversarial learning so as to solve the problem of inconsistent data distribution between the source and target domains due to crossed cameras. To address the problem of large differences in identity recognition due to camera viewpoint changes, Wang et al. [28] utilised a camera-aware Proxies method based on camera-awareness and designed intra-camera and inter-camera contrast learning components to effectively improve intra-camera and inter-camera identity recognition. Luo et al. [29] used a camera-aware approach to constrain the commonly used neighbourhood invariance method to supervise the feature learning of the target domain identity to bridge the gap between the source and target domains. The camera-awareness-based approach is robust to camera changes but is somewhat limited by the lack of paired true label information across camera samples.

**The third approach** is based on contrast learning. Contrast learning does not require the use of a fully connected network layer for category mapping but only aids the calculation of classification losses within a larger training batch or with the help of external category feature storage units. In recent times, the combination of clustering or KNN nearest neighbour based methods and contrast learning has led to the acquisition of more valid positive samples, thus making contrast learning more effective in unsupervised representation learning tasks. Zhong et al. [30] selected valid positive instances through learning sample invariance, camera invariance and nearest neighbour invariance. Yang et al. [31] proposed a semi-supervised contrast-learning approach. For labelled images, the distance to the cluster centres is reduced, and the distance to the other cluster centres and unlabelled images is increased. Moreover, for unlabelled images, the distance to the nearest neighbours is reduced, and the distance to the rest of the images is increased. Ge et al. [32] designed a hybrid external storage unit for contrast learning using the clustering centre features of the source domain data, the clustering centre features of the target domain data and the features of the unclustered instances of the target domain data. Although the features between the three different categories were considered, the updating process of the storage unit was severely affected, and there was also a failure to consider the relationship between multiple samples at the instance level. So, based on this paper, improvements have been made.

## 3. Methods

In this section, we first introduce the overall structure of our proposed network model, then briefly describe the preliminary elements of unsupervised person re-identification and finally detail the specific details of each module.

### 3.1. Overview

**Figure 2:**
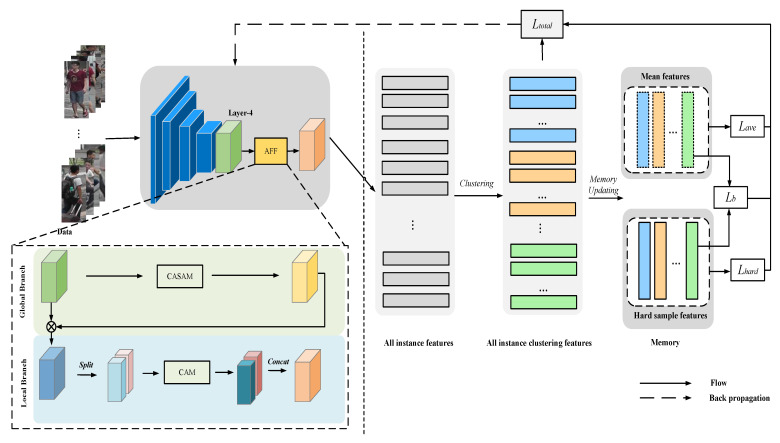
Our AFF_SCL framework for the unsupervised person Re-ID pipeline. It consists of two main stages: The left side of the dashed line represents our proposed the AFF module, including the interaction of global and local branches; The right side of the dashed line represents the SCL module, including the mean feature contrast loss, the hard sample contrast loss and a balancing loss.

To address the problem in both stages of the unsupervised person-re-identification task, we propose a framework based on attention-guided fine-grained feature-network and symmetric-contrast learning (AFF_SCL), as shown in Figure 2.

In the first stage, inspired by the feature pyramid [10], we combine fine-grained features and two attention mechanisms to propose an attention-based fine-grained feature network (AFF) that guides the network to mine feature information from different granularities to achieve generalised and distinguishable representations, further improving the neural network’s ability to extract more distinguishable features. As shown on the left in Figure 2, we remove the average pooling layer and the fully connected layer after Layer-4 on the basis of the ResNet-50 baseline, and remove the down-sampling operation to obtain the global features of the people. In the global branch, in order to further capture the overall information of the image, the global features are processed by the hybrid attention module (CASAM) to focus on the people information of the whole image and reduce the interference from the background, and then after attention processing the features are fused with the original image to obtain the intermediate features. In the local branch, the intermediate features are split from the channel dimension to obtain two local features, each of which is processed by the channel-attention module (CAM) to explore the deep semantic information, and then the features after the attention processing are stitched together to obtain the final people-feature representation.

As shown on the right in Figure 2, in the second stage, our proposed symmetric contrast-learning (SCL) method based on mean samples and hard samples uses a hybrid loss function to jointly construct a complete person-re-identification model for unsupervised scenarios. The unlabelled pedestrian data in the target domain are fed into the AFF model to extract features and, after obtaining all the target domain instance features the DBSCAN clustering algorithm, is used to analyse the data distribution and obtain different clusters, generating a pseudo label for each class as the identity of that class. When selecting class representatives from each class, mean features and hard sample features are used to perform momentum with the help of storage units. Finally, a symmetric contrast loss is designed to combine the mean-feature contrast loss and the hard-sample contrast loss. The balancing loss is applied to reduce the difference between the mean feature and the hard sample feature, and the model is iteratively trained to optimize the model’s parameters.

### 3.2. Preliminary

In an unsupervised person-re-identification task, given an unlabeled training set consisting of n image samples $X=\left\{x_{1},x_{2},\cdots ,x_{n}\right\}$, an encoder programmed for extracting features from the input image learns $\Phi \left(\theta ;x_{i}\right)$ without any available annotation. The parameters of Φ are iteratively optimized using the objective function. For inference, this feature extractor can be applied to the graph library set of Nt images, $G=\left\{g_{1},\;g_{2},\cdots ,g_{\mathrm{Nt}}\right\}$ and the query set, $Q=\left\{q_{1},q_{2},\cdots ,q_{\mathrm{Nq}}\right\}$ of Nq images. During the evaluation process, the representation of the query image $\Phi \left(\theta ;q_{i}\right)$ is used to search the graph library set, and more features similar to q are searched from the graph library set G.

### 3.3. Attention-Guided Fine-Grained Feature Network

**Figure 3:**
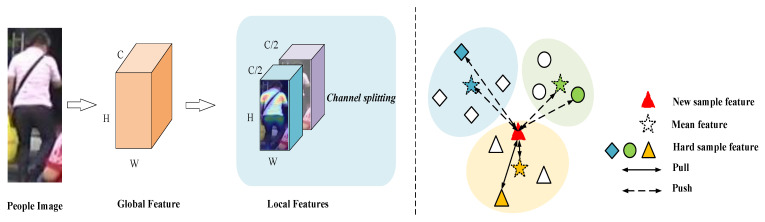
The (**left**) side of the dashed line indicates that we obtained the local features by splitting the channel equally into two blocks. The (**right**) side of the dashed line indicates the relationship between the new sample features and the different sample features.

Channel slicing allows for semantic concept-to-concept matching, which can complement the feature representation extracted from spatial chunks and also does not suffer from feature misalignment. As the CNN progresses, the final layer becomes more abstract, and the output of the channel is a more semantic feature, potentially corresponding to concepts such as hair colour, body type, etc. Therefore channel slicing has a strong advantage over spatial chunking. In this paper, for the local feature-learning method we use image channel slicing, which does not extract spatial local features of specific body parts but divides the deep features represented by the deep network in the channel dimension into two local features and forms independent channel groups. The semantic concepts of each channel group can be correlated, and local feature learning arise performed separately, as shown on the left side of the dashed line in Figure 3.

In this paper, global features mainly are processed using hybrid attention and local features, after channel segmentation is processed using channel attention.

#### 3.3.1. CASAM

For global branching, CASAM hybrid attention uses the idea of the Convolutional Block Attention Module [33], which combines channel attention and spatial attention and further refines and fuses image features by considering the multidimensional information of people images. Firstly, the image feature *F* is first obtained in the channel attention sub-module as feature $F_{C}$, then the feature *F* is multiplied by $F_{C}$ to obtain the intermediate refinement feature $F^{\prime }$ then sent to the spatial attention sub-module to obtain feature $F_{S}$, and finally $F_{S}$ is multiplied by $F^{\prime }$ to obtain the final refinement feature $F^{\prime \prime }$. The computational equation of the complete attention module can be expressed as:

(1) $$F_{C}=\sigma \left(W_{1}\left(\;W_{0}\left(\mathrm{MaxPool}\left(F\right)\right)\right)+W_{1}\left(\;W_{0}\left(\mathrm{AvgPool}\left(F\right)\right)\right)\right)$$

(2) $$F^{\prime }=F⊗F_{C}$$

(3) $$F_{S}=\sigma \left({\mathrm{conv}}^{7\times 7}\left(\left[\mathrm{MaxPool}\left(F^{\prime }\right);\mathrm{AvgPool}\left(F^{\prime }\right)\right]\right)\right)$$

(4) $$F^{\prime \prime }=F^{\prime }⊗F_{S},$$

where ⊗ denotes pixel-to-pixel multiplication, $F_{C}$ is the channel attention value, σ denotes the sigmoid function, $\;W_{0}$ and $W_{1}$ are the weights in the multi-layer perceptron, $F_{S}$ is the spatial attention value, and ${\mathrm{conv}}^{7\times 7}$ is the convolution layer of size 7 × 7.

CASAM solves the problem of heterogeneity between features by refining and merging the features extracted on the channel and spatial dimensions. For a people image, we need to focus more on the foreground information in the image, while reducing the interference of background information. The combination of channel attention and spatial attention can be a good solution to this problem and is also suitable for handling global features of people.

#### 3.3.2. CAM

For local branching, the CAM attention module uses the idea of Squeeze-and-Excitation networks, the basic idea of which is to make the network model ignore some less useful features, learn adaptively, determine the role of features according to their importance and enhance the feature channels that are useful for the task [34]. The Re-ID method requires more original information to be retained for some similar people samples, then a large amount of detailed feature information is needed to supplement it, so the CAM module is introduced to simulate the interdependencies between the features of each channel, to find more discriminative features and to improve the representation capability of the network. The CAM module consists of three components. **Squeezing**: Features are squeezed using global average pooling to transform a two-dimensional feature channel into a feature vector and use this vector to obtain information. **Excitation**: This consists of two fully connected layers and a sigmoid layer. The fully connected layer collects all the feature information, while the sigmoid layer places the input data between 0 and 1 to obtain the weights of each channel in the feature map. **Fusion**: The processed features are fused with the original image and the weights are attached to the individual channels, which obtains channels of different importance. It is expressed as:

(5) $$G_{C}=\sigma \left(W_{2}^{\prime }\mathrm{ReLU}\left(W_{1}^{\prime }\mathrm{AvgPool}\left(G\right)\right)\right)$$

(6) $$G^{\prime }=G⊗G_{C},$$

where $W_{1}^{\prime }$ and $W_{2}^{\prime }$ are the weights in the multi-layer perceptron, σ denotes the sigmoid function, and ReLU denotes the ReLU function.

The CAM module is easy to build, simple to use, relatively inexpensive to compute and can be trained directly. Therefore, the integration of the CAM module into the network architecture not only significantly improves the efficiency of the network, but also improves the importance of focusing on the channels after the people-feature segmentation, which can greatly improve the model’s ability to discriminate detailed information.

### 3.4. Symmetric Contrast Learning

To optimise the second stage of unsupervised learning and to improve the model’s ability to discriminate features, contrast learning, as a type of metric learning, is mainly used to increase the distance of negative sample pairs and decrease the distance of positive sample pairs. Inspired by the noise contrast estimation loss function or the N-pair loss function, some contrast learning loss functions have been successfully applied to unsupervised representation learning tasks in recent years.

Thus, inspired by the literature [35], instance-level hard-sample contrast loss is introduced to exploit the full feature potential of hard samples [36,37]. By hard samples, we mean samples that are very different compared to samples in the same identity, samples that are very different in different identities, and, in short, samples that are hard to distinguish from positive samples. As shown in Figure 3, calculating the clustered mean features without considering the effect of noise caused by the wrong samples optimises the trends of the features, making the same clusters more compact and enhancing identity discrimination. However, introducing hard samples and comparing the inputted new samples with hard positive samples belonging to the same clusters and hard negative samples from other clusters, and thus learning to distinguish easily confused samples, can be a good solution to the problem that similar samples cannot easily be distinguished.

Therefore, on the premise that hard samples can provide a large amount of information for training, using contrast learning improves intra-class compactness and inter-class separability, improves the final discriminative power of the neural network and speeds up the convergence of the network. In this paper, a combination of mean-feature contrast loss and hard-sample contrast loss is used to select mean features and hard sample features when selecting representative features for storage unit updates to tap into the discriminative differentiation ability of the model.

In this paper, we use a state-of-the-art contrast learning method using the InfoNCE loss function [38], as shown in Equation (7), to minimise the distance between samples of the same identity and maximise the distance between samples of different identities.

(7) $$L_{q}=-\log\frac{\exp\left(query\cdot class^{+}\right)/\tau }{\sum_{i=0}^{K}\exp\left(query,class_{i}\right)/\tau },$$

where query is a query feature, and $class^{+}$ is a positive feature that is selected from candidate classes $class^{1},class^{2},\cdots ,class^{K}$ with the same label as query. τ is a temperature hyperparameter that controls the similarity scale. Thus, by combining the mean-feature contrast loss (cluster-level loss) and the hard-sample-feature contrast loss (instance-level loss), while introducing a balancing loss, a total loss function is proposed, as shown in Equation (8).

(8) $$L_{total}=\lambda_{1}L_{hard}+\lambda_{2}L_{ave}+L_{b},$$

where λ is a balancing factor with default settings of 0.75 for $\lambda_{1}$ and 0.25 for $\lambda_{2}$. In the following sections, we will describe the above loss formulae in more detail.

#### 3.4.1. Mean-Feature Contrast Loss

Some instance-level memory-storage approaches require maintaining each instance feature of the dataset and updating the corresponding memory storage with its own instance features in each small batch, suffering from inconsistent memory-storage updates. In each training iteration, a smaller cluster may have a higher proportion of instances updated and take longer than a larger cluster due to the unbalanced distribution of cluster sizes. Different instances in the same cluster will, therefore, have different update states. Unlike previous approaches to instance-level memory storage, this paper uses mean features to reserve one representative feature for each cluster, rather than reserving all instance features. Regardless of whether the clusters are large or small, the corresponding storage units are updated, ensuring consistency in the updating of features within the same cluster. During the training process, we sample P personas and a fixed number of K instances of each persona. As a result, a total number of P × K query images is obtained in small batches of training.

The mean feature contrast loss is shown in Equation (9), where *q* is the current query sample feature, $c_{i}$ denotes the cluster centre feature of class *i*, and τ is a temperature hyperparameter.

(9) $$L_{ave}=-\log\frac{\exp\left(q\cdot c^{+}\right)/\tau }{\sum_{i=0}^{K}\exp\left(q,c_{i}\right)/\tau }$$

The clustering centroids $c_{1},c_{2},\cdots ,c_{K}$ are calculated and stored in the memory of the mean feature contrast loss, for which they are updated in the following way.

(10) $$c_{i}\leftarrow m\cdot c_{i}+\left(1-m\right)\cdot {\bar{c}}_{i},$$

where $c_{i}$ is the mean of the *i*-th class of instance characteristics in the small-batch process, and *m* is the momentum update factor.

#### 3.4.2. Hard-Sample Feature Contrast Loss

Specifically, the most difficult instance feature in each person’s identity is selected for storage and then dynamically updated for each class. The hard-sample feature contrast loss function is shown in Equation (11), where *q* is the current query sample, $q_{hard}^{i}$ is the hard sample in class *i*, and τ is a temperature hyperparameter.

(11) $$L_{hard}=-\log\frac{\exp\left(q\cdot q_{hard}^{+}\right)/\tau }{\sum_{i=0}^{K}\exp\left(q,q_{hard}^{i}\right)/\tau }$$

The feature update is shown in Equations (12) and (13).

(12) $$q_{hard}^{i}\leftarrow \underset{q}{\arg\min}q\cdot c_{i},q\in Q^{i}$$

(13) $$q_{hard}^{i}\leftarrow m\cdot q_{hard}^{i}+\left(1-m\right)\cdot {\bar{q}}_{hard}^{i},$$

where the hard sample is the instance with the least similarity to the clustering feature. We use the dot product as a measure of similarity, and *m* is the momentum update factor. $Q^{i}$ is the set of features of instances with identity *i* in the current small batch.

#### 3.4.3. Balance Loss

In the training process, in order to balance the mean features and hard sample features, a consistency-loss balancing loss $L_{b}$ based on a distance metric is used to constrain the two features generated in the storage unit prediction, reducing the imbalance between the features, while the loss also accelerates the convergence of the model and reduces the training time, as shown in Equation (14).

(14) $$L_{b}\left(x\right)=L_{b}\left(c-q_{hard}\right)=\frac{1}{4}\left\{\begin{array}{c} \frac{\alpha }{b}(b|x|+1)\ln(b|x|+1)-\alpha |x|,if|x|<1\\ \gamma |x|+\frac{\gamma }{b}-\alpha ,otherwise, \end{array}\right.$$

where α=0.9, γ=αln(b+1)=1.5 [39].

## 4. Experiments

### 4.1. Datasets and Evaluation Metrics

**Datasets** To validate the effectiveness of our proposed model, we conducted experiments on two mainstream datasets, as is shown in Table 1. **Market-1501** [40]: This contains the identities of 1501 individuals and 32,668 images generated from six camera views automatically cropped by the people detector of DPM. **DukeMTMC-reID** [41]: This is a subset of the DukeMTMC dataset. These images were generated from eight camera views by manual annotation and contain the identities of 1812 individuals and 36,411 images.

**Evaluation metrics** For Re-ID, Rank-n (n = 1, 5, 10), Cumulative Match Characteristic (CMC) curves and mean Average Precision (mAP) are commonly used as evaluation metrics to measure the performance of the algorithm. Rank-n represents the probability that all query images have matching images in the first n images in the ranking result. CMC represents the probability curve of Rank-n, whose horizontal coordinate indicates finding a match among the first n candidate images, and whose vertical coordinate represents the probability that a matching image appears among the first n candidate images. The mean average precision (mAP) indicates the average precision of the successful retrieval for all query images. It can be seen that mAP measures not only the accuracy of feature extraction, but also the accuracy of classification results and can provide a comprehensive assessment of the performance of the person-re-identification algorithm.

### 4.2. Implementation Details

In this section, experiments will be implemented on two datasets, Market-1501 and DukeMTMC-reID. Features are extracted using the AFF network, and the model is initialised using parameters pre-trained on ImageNet. For the training stage, pre-processing operations such as random erasure were performed on the training images. The image size was set to 256 × 128, and the size of each batch was set to 128 images containing 16 pedestrian identities, with 8 instances drawn for each person. The initial learning rate was set to $3.5\times 10^{-4}$ and reduced to 1/10 of the previous value every 20 epochs out of a total of 60 epochs. The optimiser was set to Adam optimiser, and the training weights were decayed to $5\times 10^{-4}$. Clustering was performed using DBSCAN, with a clustering hyperparameter minPts of 4, a Jaccard distance hyperparameter K1 value of 30 and a K2 value of 6. Experiments were performed in Pytorch1.7, and two NVIDIA TITAN RTX GPUs were trained and tested.

### 4.3. Comparison with Existing Methods

In this section, we will compare the two settings from USL and UDA with the existing methods.

The USL method corresponds to the fully unsupervised person re-identification in the research line. Such methods rely entirely on the discriminatory ability of the pedestrian features extracted by the model and are also influenced by the performance of the clustering algorithm in the unsupervised learning stage. Therefore, the method in this paper addresses these two aspects for improved research, and the superiority of the method in this paper can be seen in Table 2.

Earlier approaches such as BUC [5] and DBC [42] improve on clustering methods by ignoring the uniqueness represented by the features, and the identification of pedestrian identities can greatly affect the clustering effect; MMCL [43] and JVTC [44] use multi-label classification, which undoubtedly increases the complexity of the model and also ignores the optimisation of the model’s ability to extract features. In contrast to the SpCL [32] approach, which uses the contrast learning framework, all instances of the target domain are put into the storage unit when storing features and only rely on the mean features to guide the model, increasing the probability of introducing noisy samples into the model.

Therefore, as can be seen from Table 2, compared with other excellent algorithms, the method in this paper also has certain superiority. mAP and Rank-1 reached 78.8% and 90.9% on the Market-1501 dataset and 68.6% and 82.4% on the DukeMTMC-reID dataset, fully verifying the proposed validity and rationality of the proposed idea.

We also compare our method with some existing UDA person-re-identification method, which makes full use of the labelled source-domain dataset, by adding the labelled source domain data to the training along with the unlabelled target domain data when training the model and testing it on the target domain dataset. As a result, better results are usually achieved than those achieved with the USL person-re-identification method. The symmetric contrast loss can also be easily generalised to the UDA person-re-identification method. However, as we do not have a feature-enhancement method such as image-style migration for the source domain dataset, we are not concerned with how to use the labelled source-domain dataset, and the labelled source dataset is not very helpful. Table 3 shows that, even with more training data, our pure unsupervised person-re-identification method still has a higher mapping than the UDA person-re-identification method.

### 4.4. Ablation Studies

**Impact of Loss Function.** According to the method proposed in this paper for the selection of clustering features when selecting different features to design different loss perspectives, this paper tested four different losses. The experimental results are shown in Table 4. It can be seen that this paper’s proposed combination of mean feature contrast loss and hard sample contrast loss method has advantages.

**Impact of Hyper-Parameter λ.** λ is the balancing factor, which plays an important role in influencing the weights of the mean sample loss and the hard-sample loss. $\lambda_{1}$ denotes the hard-sample loss parameter and $\lambda_{2}$ denotes the clustering mean-feature loss parameter.

As can be seen from Table 5, combining these two contrasting losses and increasing the diversity within the class clearly results in better performance. In addition, when $\lambda_{1}$ = 0.75 and $\lambda_{2}$ = 0.25, the experiment obtained the best performance in mAP by 78.8%, which shows that the information of hard samples can help a lot in the improvement of unsupervised clustering, and it can also be seen that the balance loss can effectively regulate the differences between features. Therefore, the symmetric-contrast learning method proposed in this paper has significant advantages over other methods in the training process.

**Figure 4:**
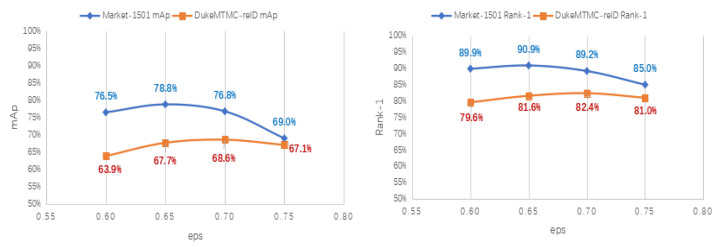
The result of mAP and Rank-1 on Market1501 and DukeMTMC-reID.

**Impact of eps.** eps is a parameter within the clustering algorithm DBSCAN representing the radius of the clusters selected for clustering, which affects the final number of clusters. If the value of d is chosen to be too small, a larger portion of the data will be considered to be outliers. If too large a value is chosen, the clusters will be merged, and most of the data points will be located in the same cluster. Ultimately, it will affect the performance of the algorithm. We analysed the impact of d on the performance of the Market-1501 and DukeMTMC-reID datasets. We can see from Figure 4 that the value of d has a relatively large impact on the results, and the value of d for the optimal results is different for different datasets. The best performance is achieved on the Market-1501 dataset when d = 0.65. The best performance on the DukeMTMC-reID dataset is achieved when d = 0.7.

**Impact of Batch Size and k.** In Table 6, ‘Batch size’ denotes the batch size, and k denotes the number of instance samples per identity sampled, from which it can be seen that, as the Batch size and k increase proportionally, the performance of the model increases further, which can also reflect from the side that increasing the diversity of samples has a great effect on improving the discriminative power of the model. Due to the GPU memory, this experiment validates that the upper limit of Batch size is 256.

**Impact of AFF and Model Size Analysis.** As it involves the processing of features, in order to fully demonstrate the effectiveness of the AFF module, we embed the module into the supervised model, using a reid-strong-baseline. The best results are achieved by using CASAM attention for **Local** branches and CAM attention for **Global** branches. It can also be seen in Table 7 that the coarse-grained to fine-grained feature-fusion approach has better performance, because it first focuses on pedestrian features from global features to reduce the interference of other factors such as background, and then the local segmentation does channel attention, which can focus on the semantic information of some deep features, such as backpacks, hair, etc. This semantic information plays a key role in improving the model’s performance, further proving that the AFF model can handle the Re-ID task in different scenarios.

We also analysed the size of the model, and we used floating-point operations (FLOPs), parameters (Params) and memory to illustrate the space and time complexity of our model and some related models, and the results are listed in Table 8. Compared to the multi-branch network MGN [3], our approach has close memory but substantially fewer parameters and reduced FLOPs. MHN [11] requires the computation of complex higher-order attention distributions, so its FLOPs are almost more than twice as large as ours. As can be seen from the table, the more branches the model has, the larger the number of parameters and the amount of computation the model has. It is thus introduced that the size of the model in this paper meets the requirement of a lightweight and practical model for practical application scenarios and has some practicality.

## 5. Discussion

In the latest literature review [49], it is elaborated that, in most of the current research on unsupervised person-re-identification tasks, basically only one stage in the task is improved, which is one of the pain points that needs to be addressed urgently, so this paper proposes the AFF_SCL framework based on this, and the two stages are improved separately. Multiple attention methods are applied in the AFF model, and a hybrid loss is designed in the SCL method. Although the accuracy of the model surpasses that of the single-stage improvement method, it is certainly a little more complex in terms of the size and computation of the model than the single-stage improvement method.

Therefore, in future work, we will focus on how to use a lightweight and powerful feature extractor in the feature-extraction stage and how to reduce the difference between the target and source domains and the reliance on annotated data in the unsupervised learning stage, which is more suitable for real industrial scenarios.

## 6. Conclusions

In this paper’s work, we have made improvements in both stages of the unsupervised person-re-identification task simultaneously. In the first stage, an attention-guided fine-grained feature network (AFF) is proposed to achieve a generalised and distinguishable feature representation by mining global and local feature information, using attention-focused semantic information, while migrating the module to a supervised baseline, confirming its validity. In the second stage, a symmetric contrast learning (SCL) module is proposed to replace the traditional loss function to fully exploit the potential of the sample information. Finally, this unsupervised person-re-identification framework (AFF_SCL) is tested on the Market-1501 and DukeMTMC-reID datasets, and the experiments demonstrate that the framework can be used for the multi-scene task of person re-identification with certain superiority and generalisation capability.

## Figures and Tables

**Table 1:** The detailed information of datasets used in the paper.

Dataset	Train Sets (IDs/Images)	Test Sets (IDs/Images)	Query Images	Cameras	Total Images
Market-1501 [40]	751/12,936	750/19,732	3368	6	32,668
DukeMTMC-reID [41]	702/16,522	702/19,889	2228	8	36,441

**Table 2:** Comparison of USL methods on Market1501 and DukeMTMC-reID.

Methods	Year	Market-1501		DukeMTMC-reID	
		mAP	Rank-1	mAP	Rank-1
SSL [45]	2020	37.8	71.7	28.6	52.5
BUC [5]	2019	38.3	66.2	27.5	47.4
DBC [42]	2019	41.3	69.2	30.0	51.5
MMCL [43]	2020	45.5	80.3	40.2	65.2
JVTC [44]	2020	41.8	72.9	42.2	67.6
HCT [6]	2020	56.4	80.0	50.7	69.6
DSCE [16]	2021	61.7	83.9	53.8	73.8
CycAs [46]	2020	64.8	84.8	60.1	77.9
IICS [18]	2021	72.9	89.5	64.4	80.0
SpCL [32]	2020	73.1	88.1	65.3	81.2
**Ours**	-	**78.8**	**90.9**	**68.6**	**82.4**

**Table 3:** Comparison of UDA methods on Market1501 and DukeMTMC-reID.

Methods	Year	D → M		M → D	
		mAP	Rank-1	mAP	Rank-1
SSG [15]	2019	58.3	80.0	53.4	73.0
AE [7]	2020	58.0	81.6	46.7	67.9
MMT [21]	2020	65.1	78.0	71.2	87.7
DAAL [23]	2020	67.8	86.4	63.9	77.6
GPR [29]	2020	71.5	88.1	65.2	79.5
MEB-Net [22]	2020	76.0	89.0	66.1	79.6
**Ours**	-	**77.7**	**91.0**	**66.5**	**80.7**

**Table 4:** Impact of Loss Function on Market1501.

Loss	Market-1501	
	mAP	Rank-1
Baseline loss (cross-entropy + triples)	55.8	75.3
Mean feature contrast loss	70.6	86.9
Hard sample contrast loss	76.3	89.3
**Total loss**	**78.8**	**90.9**

**Table 5:** Impact of Hyper-Parameter λ on Market1501.

$\lambda_{1}$	$\lambda_{2}$	Market-1501	
		mAP	Rank-1
0	1	68.5	85.4
0.25	0.75	70.4	86.0
0.5	0.5	75.7	88.9
**0.75**	**0.25**	**78.8**	**90.9**
1	0	73.7	87.9

**Table 6:** Impact of Batch Size and k on Market1501.

Batch Size	k	Market-1501	
		mAP	Rank-1
64	4	75.6	89.0
128	8	78.8	90.9
256	16	79.6	91.4

**Table 7:** Impact of AFF on Market1501.

Methods	Year	Market-1501			
		mAP	Rank-1	Rank-5	Rank-10
Baseline ^1^	2019	81.7	92.0	-	-
MGN [3]	2018	86.9	**95.7**	98.3	**99.0**
HPM [4]	2019	82.7	94.2	97.5	98.5
MHN-6(PCB) [11]	2019	85.0	95.1	98.1	98.9
HOReID [47]	2020	84.9	94.2	-	-
GRL [48]	2021	80.5	91.7	-	-
Baseline + L	-	86.8	94.5	**98.4**	**99.0**
Baseline + G	-	87.4	94.9	98.1	98.9
**Baseline + AFF**	-	**88.2**	95.0	98.3	98.9

^1^https://github.com/michuanhaohao/reid-strong-baseline.

**Table 8:** Model Size Analysis. The unit of Params is M, the unit of Memory is MB, and the unit of FLOPs is G.

Methods	Params	FLOPs	Memory
MHN-6(PCB) [11]	30.36	24.55	186.18
MGN [3]	74.38	11.94	179.55
Ours	26.70	9.17	186.26

## Data Availability

Not applicable.

## References

[1] Zheng L., Yang Y., Hauptmann A.G. (2016). Person Re-identification: Past, Present and Future. arXiv.

[2] Wei W., Yang W., Zuo E., Qian Y., Wang L. (2022). Person Re-Identification Based on Deep Learning—An Overview. J. Vis. Commun. Image Represent..

[3] Wang G., Yuan Y., Chen X., Li J., Zhou X. Learning Discriminative Features with Multiple Granularities for Person Re-Identification. Proceedings of the 2018 26th ACM International Conference on Multimedia.

[4] Fu Y., Wei Y., Zhou Y., Shi H., Huang G., Wang X., Yao Z., Huang T. Horizontal Pyramid Matching for Person Re-Identification. Proceedings of the AAAI Conference on Artificial Intelligence.

[5] Lin Y., Dong X., Zheng L., Yan Y., Yang Y. A Bottom-Up Clustering Approach to Unsupervised Person Re-Identification. Proceedings of the 2019 AAAI Conference on Artificial Intelligence.

[6] Zeng K. Hierarchical Clustering with Hard-batch Triplet Loss for Person Re-identification. Proceedings of the 2020 IEEE Conference on Computer Vision and Pattern Recognition (CVPR).

[7] Ding Y., Fan H., Xu M., Yang Y. (2020). Adaptive Exploration for Unsupervised Person Re-Identification. ACM Trans. Multimed. Comput. Commun. Appl..

[8] Sun Y., Zheng L., Yang Y., Tian Q., Wang S. Beyond Part Models: Person Retrieval with Refined Part Pooling (and a Strong Convolutional Baseline). Proceedings of the European Conference on Computer Vision (ECCV).

[9] Chen H., Lagadec B., Bremond F. Learning Discriminative and Generalizable Representations by Spatial-Channel Partition for Person Re-Identification. Proceedings of the 2020 IEEE Winter Conference on Applications of Computer Vision (WACV).

[10] Chen G., Gu T., Lu J., Bao J.A., Zhou J. (2021). Person Re-identification via Attention Pyramid. IEEE Trans. Image Process..

[11] Chen B., Deng W., Hu J. Mixed High-Order Attention Network for Person Re-Identification. Proceedings of the IEEE/CVF International Conference on Computer Vision.

[12] Liu X., Zhao H., Tian M., Sheng L., Shao J., Yi S., Yan J., Wang X. HydraPlus-Net: Attentive Deep Features for Pedestrian Analysis. Proceedings of the 2017 IEEE International Conference on Computer Vision (ICCV).

[13] Fan H., Zheng L., Yan C., Yang Y. (2018). Unsupervised Person Re-identification: Clustering and Fine-tuning. ACM Trans. Multimed. Comput. Commun. Appl..

[14] Wu J., Liao S., Lei Z., Wang X., Yang Y., Li S.Z. Clustering and Dynamic Sampling Based Unsupervised Domain Adaptation for Person Re-Identification. Proceedings of the 2019 IEEE International Conference on Multimedia and Expo (ICME).

[15] Fu Y., Wei Y., Wang G., Zhou Y., Shi H., Uiuc U., Huang T. Self-Similarity Grouping: A Simple Unsupervised Cross Domain Adaptation Approach for Person Re-Identification. Proceedings of the 2019 IEEE/CVF International Conference on Computer Vision (ICCV).

[16] Yang F., Zhong Z., Luo Z., Cai Y., Lin Y., Li S., Sebe N. Joint Noise-Tolerant Learning and Meta Camera Shift Adaptation for Unsupervised Person Re-Identification. Proceedings of the IEEE/CVF Conference on Computer Vision and Pattern Recognition (CVPR).

[17] Yang Q., Yu H.X., Wu A., Zheng W.S. Patch-Based Discriminative Feature Learning for Unsupervised Person Re-Identification. Proceedings of the 2019 IEEE/CVF Conference on Computer Vision and Pattern Recognition (CVPR).

[18] Xuan S., Zhang S. Intra-Inter Camera Similarity for Unsupervised Person Re-Identification. Proceedings of the IEEE Conference on Computer Vision and Pattern Recognition (CVPR).

[19] Long M., Cao Y., Wang J., Jordan M.I. Learning Transferable Features with Deep Adaptation Networks. Proceedings of the International Conference on Machine Learning, PMLR.

[20] Peng P., Xiang T., Wang Y., Pontil M., Gong S., Huang T., Tian Y. Unsupervised Cross-Dataset Transfer Learning for Person Re-identification. Proceedings of the 2016 IEEE Conference on Computer Vision and Pattern Recognition (CVPR).

[21] Ge Y., Chen D., Li H. Mutual Mean-teaching: Pseudo Label Refinery For Unsupervised Domain Adaptation On Person Re-identification. Proceedings of the 2020 International Conference on Learning Representations (ICLR).

[22] Zhai Y., Ye Q., Lu S., Jia M., Ji R., Tian Y. (2020). Multiple Expert Brainstorming for Domain Adaptive Person Re-identification. arXiv.

[23] Huang Y., Peng P., Jin Y., Li Y., Xing J. Domain Adaptive Attention Learning for Unsupervised Person Re-Identification. Proceedings of the AAAI Conference on Artificial Intelligence.

[24] Wei L., Zhang S., Gao W., Tian Q. Person Transfer GAN to Bridge Domain Gap for Person Re-identification. Proceedings of the 2018 IEEE/CVF Conference on Computer Vision and Pattern Recognition (CVPR).

[25] Deng W., Zheng L., Ye Q., Kang G., Yang Y., Jiao J. Image-Image Domain Adaptation with Preserved Self-Similarity and Domain-Dissimilarity for Person Re-identification. Proceedings of the IEEE/CVF Conference on Computer Vision and Pattern Recognition (CVPR).

[26] Chen Y., Zhu X., Gong S. Instance-Guided Context Rendering for Cross-Domain Person Re-Identification. Proceedings of the 2019 IEEE/CVF International Conference on Computer Vision (ICCV).

[27] Qi L., Wang L., Huo J., Zhou L., Shi Y., Gao Y. A Novel Unsupervised Camera-Aware Domain Adaptation Framework for Person Re-Identification. Proceedings of the 2019 IEEE/CVF International Conference on Computer Vision (ICCV).

[28] Wang M., Lai B., Huang J., Gong X., Hua X.S. (2020). Camera-Aware Proxies for Unsupervised Person Re-Identification. arXiv.

[29] Luo C., Song C., Zhang Z. (2020). Generalizing Person Re-Identification by Camera-Aware Invariance Learning and Cross-Domain Mixup. Proceedings of the European Conference on Computer Vision (ECCV).

[30] Zhong Z., Zheng L., Luo Z., Li S., Yang Y. Invariance Matters: Exemplar Memory for Domain Adaptive Person Re-Identification. Proceedings of the 2019 IEEE/CVF Conference on Computer Vision and Pattern Recognition (CVPR).

[31] Yang Q., Wu A., Zheng W.S. Deep SemiSupervised Person ReIdentification with External Memory. Proceedings of the 2019 IEEE International Conference on Multimedia and Expo (ICME).

[32] Ge Y., Zhu F., Chen D., Zhao R., Li H. Self-Paced Contrastive Learning with Hybrid Memory for Domain Adaptive Object Re-ID. Proceedings of the Neural Information Processing Systems.

[33] Woo S., Park J., Lee J.Y., Kweon I.S., Ferrari V., Hebert M., Sminchisescu C., Weiss Y. (2018). CBAM: Convolutional Block Attention Module. Proceedings of the Computer Vision—ECCV 2018.

[34] Hu J., Shen L., Albanie S., Sun G. Squeeze-and-Excitation Networks. Proceedings of the IEEE Conference on Computer Vision and Pattern Recognition (CVPR).

[35] Dai Z., Wang G., Yuan W., Liu X., Zhu S., Tan P. (2021). Cluster Contrast for Unsupervised Person Re-Identification. arXiv.

[36] Hu Z., Zhu C., He G. Hard-Sample Guided Hybrid Contrast Learning for Unsupervised Person Re-Identification. Proceedings of the 7th IEEE International Conference on Network Intelligence and Digital Content (IC-NIDC).

[37] Yao H., Xu C. (2021). Dual Cluster Contrastive Learning for Person Re-Identification. arXiv.

[38] van den Oord A., Li Y., Vinyals O. (2018). Representation Learning with Contrastive Predictive Coding. arXiv.

[39] Pang J., Chen K., Shi J., Feng H., Ouyang W., Lin D. Libra R-CNN: Towards Balanced Learning for Object Detection. Proceedings of the 2019 IEEE/CVF Conference on Computer Vision and Pattern Recognition (CVPR).

[40] Zheng L., Shen L., Tian L., Wang S., Wang J., Tian Q. Scalable Person Re-identification: A Benchmark. Proceedings of the 2015 IEEE International Conference on Computer Vision (ICCV).

[41] Ristani E., Solera F., Zou R.S., Cucchiara R., Tomasi C. Performance Measures and a Data Set for Multi-Target, Multi-Camera Tracking. Proceedings of the European Conference on Computer Vision (ECCV).

[42] Ding G., Khan S., Tang Z., Zhang J., Porikli F. (2019). Towards Better Validity: Dispersion Based Clustering for Unsupervised Person Re-identification. arXiv.

[43] Wang D., Zhang S. Unsupervised Person Re-Identification via Multi-Label Classification. Proceedings of the 2020 IEEE/CVF Conference on Computer Vision and Pattern Recognition (CVPR).

[44] Li J., Zhang S. Joint Visual and Temporal Consistency for Unsupervised Domain Adaptive Person Re-Identification. Proceedings of the European Conference on Computer Vision (ECCV).

[45] Lin Y., Xie L., Wu Y., Yan C., Tian Q. Unsupervised Person Re-identification via Softened Similarity Learning. Proceedings of the IEEE/CVF Conference on Computer Vision and Pattern Recognition (CVPR).

[46] Wang Z., Zhang J., Zheng L., Liu Y., Sun Y., Li Y., Wang S. (2022). CycAs: Self-supervised Cycle Association for Learning Re-identifiable Descriptions. Proceedings of the European Conference on Computer Vision (ECCV).

[47] Wang G., Yang S., Liu H., Wang Z., Yang Y., Wang S., Yu G., Zhou E., Sun J. High-Order Information Matters: Learning Relation and Topology for Occluded Person Re-Identification. Proceedings of the IEEE/CVF Conference on Computer Vision and Pattern Recognition (CVPR).

[48] Wang H., Bi X. (2021). Person Re-Identification Based on Graph Relation Learning. Neural Process. Lett..

[49] Ye M., Shen J., Lin G., Xiang T., Shao L., Hoi S.C.H. (2021). Deep Learning for Person Re-identification: A Survey and Outlook. IEEE Trans. Pattern Anal. Mach. Intell..

